# Pain prevalence and pain relief in end-of-life care – a national registry study

**DOI:** 10.1186/s12904-024-01497-1

**Published:** 2024-07-15

**Authors:** Christel Hedman, Per Fürst, Peter Strang, Maria E.C. Schelin, Staffan Lundström, Lisa Martinsson

**Affiliations:** 1 Department of Molecular Medicine and Surgery, Karolinska Institutet, Karolinska University Hospital, Solna (L1:00), Stockholm, SE-171 76 Sweden; 2 R & D Department, Stockholms Sjukhem Foundation, Mariebergsgatan 22, Stockholm, SE-112 19 Sweden; 3 Department of Clinical Sciences Lund, Lund University, BMC I12, Lund, SE-221 84 Sweden; 4 Department of Neurobiology, Care Sciences and Society (NVS), Division of Clinical Geriatrics, Karolinska Institutet, Blickagången 16, Huddinge, SE-141 83 Sweden; 5 Department of Oncology-Pathology, Karolinska Institutet, Anna Steckséns gata 30A, Stockholm, D2:04, SE-171 64 Sweden; 6 Department of Diagnostics and Intervention Oncology, Umeå University, Umeå, SE-901 87 Sweden

**Keywords:** Palliative care, End-of-life care, Cancer, Dementia, COPD, Hearth failure, Pain, Symptom management

## Abstract

**Background:**

Despite pain control being a top priority in end-of-life care, pain continues to be a troublesome symptom and comprehensive data on pain prevalence and pain relief in patients with different diagnoses are scarce.

**Methods:**

The Swedish Register of Palliative Care (SRPC) was used to retrieve data from 2011 to 2022 about pain during the last week of life. Data were collected regarding occurrence of pain, whether pain was relieved and occurrence of severe pain, to examine if pain differed between patients with cancer, heart failure, chronic obstructive pulmonary disease (COPD) and dementia. Binary logistic regression models adjusted for sex and age were used.

**Results:**

A total of 315 000 patients were included in the study. Pain during the last week of life was more commonly seen in cancer (81%) than in dementia (69%), heart failure (68%) or COPD (57%), also when controlled for age and sex, *p* < 0.001. Severe forms of pain were registered in 35% in patients with cancer, and in 17–21% in non-cancer patients. Complete pain relief (regardless of pain intensity) was achieved in 73–87% of those who experienced pain, depending on diagnosis. The proportion of patients with complete or partial pain relief was 99.8% for the whole group.

**Conclusions:**

The occurrence of pain, including severe pain, was less common in patients with heart failure, COPD or dementia, compared to patients with cancer. Compared with cancer, pain was more often fully relieved for patients with dementia, but less often in heart failure and COPD. As severe pain was seen in about a third of the cancer patients, the study still underlines the need for better pain management in the imminently dying.

**Trial registration:**

No trial registration was made as all patients were deceased and all data were retrieved from The Swedish Register of Palliative Care database.

## Background

Pain is one of the most feared symptoms among patients during end-of-life and effective pain control is universally considered an essential part of a good death [1, 2]. Thus, pain relief is of uttermost importance not only for patients but also their families [3].

Palliative care has traditionally focused on patients with cancer due to high level of symptom burden and for historical reasons [4]. Compared to cancer, a palliative care approach in non-malignant diseases such as chronic obstructive pulmonary disease (COPD) and heart failure has not shown as good effect on symptom control but seems to increase quality of life and decrease hospitalizations [5, 6]. Non-malignant diagnoses that in end of life have a high burden of symptoms are now increasing in palliative care [7–10].

Despite the findings that both patients with cancer and non-cancer diseases have considerable palliative care needs, there are indications that the experience of pain differs between the patient groups [11]. A review shows that in patients with in advanced, metastatic or terminal cancer disease, the pain prevalence was 55% [12] and both the prevalence and intensity of pain was increasing toward end-of-life [13] and was shown by Elmstedt et al. to reach 80% [14]. In one study reporting pain during various timepoints in the last 6 months of life, patients with cardiovascular and respiratory diseases had higher prevalence of pain (57% and 58% respectively), compared to those with neurological diseases, including dementia, with a prevalence of 43% [15]. One study showed that among all patients during their last week of life, 68% experienced pain, at least at times [16]. Although various studies describe pain in end-of-life care in different settings, a general picture about pain prevalence in various diagnoses and care settings are still partly missing. Moreover, prevalence studies concerning the last week of life have rarely been published, mainly for methodological reasons.

Knowledge about pain prevalence is important, but data on achieved pain relief during end of life is equally important since pain relief is of highest importance to patients [1]. Moreover, for patients, treatment of background pain and breakthrough of pain could be equally important, as pain, even though short term, can be highly distressing [17]. Although pain treatment is improving and the prevalence of pain among patients with cancer is slightly declining [12], as many as 20% of patients receiving home care have been reporting severe pain during the last month of life [18]. Women generally report more pain than men [19], and there are indications that this also applies to end of life [18], but how sex correlates to pain in different diagnoses is still unclear.

## Aim

The aim of this study was to examine whether pain (occurrence of pain, pain relief, and occurrence of severe pain) during the last week in life, irrespective of care setting, differ between patients with cancer, heart failure, COPD, or dementia, adjusted for sex and age.

## Methods

The working method of the Swedish Register of Palliative Care (SRPC) has previously been described [20]. In short, the health care staff fills out the web-based questionnaire retrospectively after a patient has died. Symptoms are assessed by health care professionals and are based on personal knowledge about the patient and, when possible, on documented pain assessments (e.g., Visual Analogue Scale (VAS), Numeric Rating Scale (NRS), Integrated Patient care Outcome Scale (IPOS) or Edmonton Symptom Assessment Scale, (ESAS)). Most common places of deaths in the database are hospitals wards, specialized palliative care, and residential care homes including short-term stays. Cause of death data are regularly collected from the Cause of Death registry at the National Board of Health and Welfare and are merged with data from the SRPC. Of all deaths in Sweden during 2011–2022, approximately 60% were registered in the SRPC.

In our study, all adults reported to the Swedish Register of Palliative Care (SRPC) between 2011 and 2022 who had died from cancer, COPD, heart failure or dementia, irrespective of place of care or residence, were identified. The following diagnoses according to the International Classification of Diseases 10 (ICD-10) were included: cancer – C00-C97, heart failure – I50*, COPD – J44*, dementia – F00*– F03*, G30*.

Data about pain were collected by occurrence of pain, degree of pain relief, and occurrence of severe pain, (defined as e.g. VAS/NRS > 6 or corresponding pain level using other methods), during the last week in life.

The question used to collect data about pain was “Did the person display breakthrough of any of the following symptoms at any time during the last week of life?”, with pain as one of six studied symptoms. “Yes”, “No” and “Don’t know” were the response options. Minor linguistic variations were used during the years 2011–2022.

Data about pain relief are only collected for patients who were reported to have had breakthrough of pain, and thus only those patients are included in the analysis. During the whole study period, the question about pain relief was “Pain was relieved:” with Completely/Partly/Not at all, as alternatives. In 2022, a “Don´t know” alternative to the question of pain relief was added.

During 2011 to 2017, the question about severe pain was phrased “Did the person experience severe pain at any time during the last week of life (e.g. VAS or NRS > 6 or severe pain according to another validated tool)?” with Yes/No/Don’t know, as alternatives. From 2018, there was a minor modification in the wording, and the question was omitted from the register’s data collection from 2022 and onwards.

Data was collected about sex, age and whether the patient had a parenteral opioid prescribed as needed at time of death.

During most of the study period (2011 to 2021), symptom data were not collected about patients whose deaths were not anticipated based on the actual disease trajectory, and thus these patients were excluded from analysis.

## Statistical analysis

The pain variable was dichotomized into Yes/No and the pain relief variable was dichotomized into Completely vs. Partly/Not at all. Further, the severe pain variable was dichotomized into Yes/No. Don’t know-answers were excluded in the analyses. Binary logistic regression models were created, with occurrence of pain, pain relief, and occurrence of severe pain as dependent variables, and diagnosis (cancer, heart failure, COPD or dementia) as the independent variable, adjusted for sex (as a binary variable) and age (as a continuous variable). A p-value < 0.05 was considered as statistically significant.

Analyses were performed using IBM SPSS Statistics version 28.0.1.1 [14].

## Results

### Demographics

**Fig. 1:**
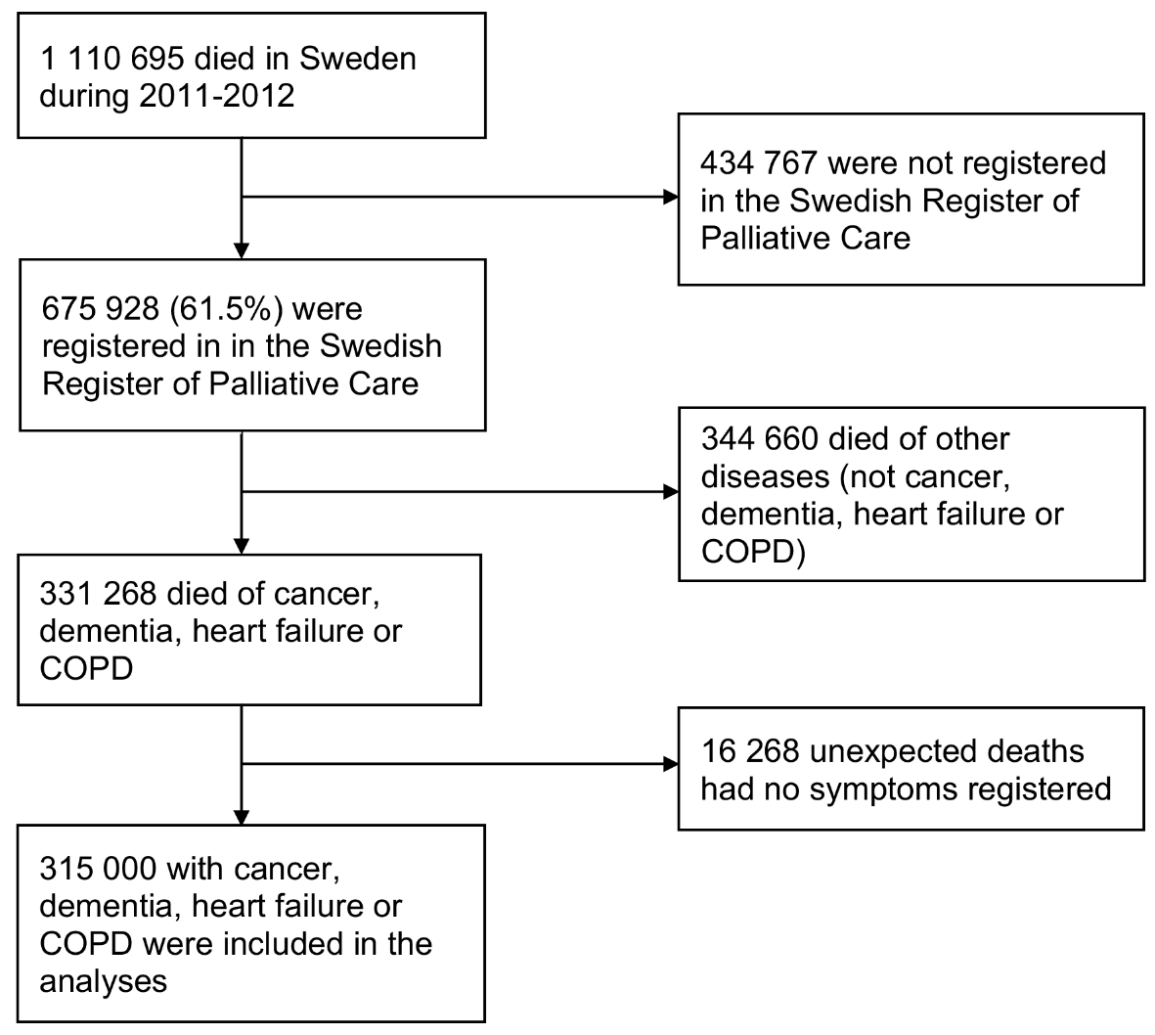
Description of which persons were included in the study

A total of 331 268 adult patients who died from cancer, heart failure, COPD, or dementia during 2011 to 2022 were identified in the database. In 16 268 patients, symptom data were not collected, and they were therefore excluded from the analyses (Fig. 1). A description of the 315 000 patients remaining in the analyses is shown in Table 1. Death occurred in acute hospitals (65 500 patients), residential care homes (129 439 patients), specialised palliative inpatient care (64 054 patients), own home (54 535) and other places (1472). Out of those who died in their own home, approximately 34 000 received specialized palliative home care.

**Table 1:** Description of the included patients regarding sex and age, divided into diagnoses and total*

		Cancer	Heart failure	COPD	Dementia	Total
Sex	Women	98 198 (48.7%)	14 112 (59.8%)	9675 (57.9%)	48 607 (66.6%)	170 592 (54.2%)
	Men	103 499 (51.3%)	9498 (40.2%)	7040 (42.1%)	24 371 (33.4%)	144 408 (45.8%)
	Total	201 697	23 610	16 715	72 978	315 000
Age (years)	Range	18–109	28–109	44–104	41–110	18–110
	Mean	75	88	81	87	79

* Included patients are divided into groups of diagnoses (cancer, heart failure, COPD and dementia) and total

### Occurrence of pain

Occurrence of pain during the last week in life was more common for patients with cancer compared to heart failure, COPD and dementia (Table 2). This was significant both in the unadjusted and the adjusted logistic regression model (Table 3), *p* < 0.001 in both models. For 9623 patients, the question about pain was answered with “Don’t know” and these were excluded from the analysis.

**Table 2:** Number and proportion of patients with occurrence of pain during the last week in life*

	Pain during last week in life	No pain during last week in life	Total
Cancer	160 690 (81.4%)	36 834 (18.6%)	197 524
Heart failure	15 084 (67.5%)	7275 (32.5%)	22 359
COPD	8711 (57.2%)	6517 (42.8%)	15 228
Dementia	48 687 (69.3%)	21 579 (30.7%)	70 266
Total	233 172 (76.4%)	72 205 (23.6%)	305 377

* Included patients are divided into groups of diagnoses (cancer, heart failure, COPD and dementia)

**Table 3:** Logistic regression model for occurrence of pain in the last week in life depending on diagnosis*

Pain during last week in life, unadjusted logistic regression model					
		p value	OR	Lower 95% CI	Upper 95% CI
Diagnosis	Cancer	Ref.			
	Heart failure	< 0.001	0.46	0.46	0.49
	COPD	< 0.001	0.31	0.30	0.32
	Dementia	< 0.001	0.52	0.51	0.53
**Pain during last week in life, logistic regression model adjusted for sex and age**					
		p value	aOR	Lower 95% CI	Upper 95% CI
Diagnosis	Cancer	Ref.			
	Heart failure	< 0.001	0.53	0.51	0.54
	COPD	< 0.001	0.32	0.31	0.33
	Dementia	< 0.001	0.56	0.55	0.58
Sex	Women	Ref.			
	Men	< 0.001	0.95	0.93	0.96
Age	Continuous (per year)	< 0.001	0.992	0.991	0.993

* Unadjusted and adjusted logistic regression

### Pain relief

In the analysis on pain relief, only patients who were reported to have had breakthrough of pain during the last week in life (*n* = 233 172) were included. Additionally, 324 patients had the answer “Don’t know” to the question about pain relief and were therefore excluded. The level of pain relief (completely, partly, or not at all) for patients who had breakthrough of pain during the last week in life, is shown in Table 4.

At time of death, 97% of cancer patients, 90% of heart failure patients, 84% of COPD patients and 94% of dementia patients had an injectable opioid prescribed against pain as needed.

**Table 4:** Pain relief (completely, partly, or not at all) for patients with pain

	Completely relieved	Partly relieved	Not at all relieved	Total
Cancer	122 121 (76.1%)	38 016 (23.7%)	364 (0.2%)	160 501
Heart failure	11 683 (77.6%)	3336 (22.2%)	41 (0.3%)	15 060
COPD	6351 (73.2%)	2292 (26.4%)	38 (0.4%)	8681
Dementia	42 200 (86.8%)	6352 (13.1%)	54 (0.1%)	48 606
Total	182 355 (78.3%)	49 996 (21.5%)	497 (0.2%)	232 848

Complete pain relief was obtained in 86.8% of patients with dementia, followed by 77.6% in heart failure, 76.1% in cancer and 73.2% in COPD (Table 4). When adjusted for age and sex, a higher OR for complete pain relief remained for patients with dementia (OR 1.76 (1.71–1.82)), whereas the OR was lower for heart failure (OR 0.92 (0.88–0.96)) and for COPD (OR 0.78 (0.74–0.82)) compared to cancer (Table 5). Almost all patients experienced at least a partial pain relief; the proportion of patients who were not relieved at all varied between 0.1 and 0.4%, depending on diagnosis.

**Table 5:** Logistic regression model for the odds of severe pain during the last week as an effect of diagnosis

Complete relief of pain during last week in life, unadjusted logistic regression model					
		p value	OR	Lower 95% CI	Upper 95% CI
Diagnosis	Cancer	Ref.			
	Heart failure	< 0.001	1.09	1.05	1.13
	COPD	< 0.001	0.86	0.82	0.90
	Dementia	< 0.001	2.07	2.01	2.13
**Complete relief of pain during last week in life, logistic regression model adjusted for sex and age**					
		p value	aOR	Lower 95% CI	Upper 95% CI
Diagnosis	Cancer	Ref.			
	Heart failure	< 0.001	0.92	0.88	0.96
	COPD	< 0.001	0.78	0.74	0.82
	Dementia	< 0.001	1.76	1.71	1.82
Sex	Women	Ref.			
	Men	< 0.001	0.89	0.87	0.90
Age	Continuous (per year)	< 0.001	1.01	1.01	1.01

### Severe pain

The question about severe pain was registered for 288 849 patients in the database, from which 35 742 were excluded due to a “Don’t know”-answer. Patients with heart failure, COPD and dementia less often experienced severe pain compared to patients with cancer (Table 6). This finding remained after adjusting for sex and age (Table 7).

**Table 6:** Number and proportions of patients with severe pain during the last week subdivided on diagnosis

	Severe pain	No severe pain	Total
Cancer	56 639 (34.8%)	106 047 (65.2%)	162 686
Heart failure	3957 (21.4%)	14 553 (78.5%)	18 510
COPD	2119 (17.4%)	10 081 (82.6%)	12 200
Dementia	11 656 (19.5%)	48 055 (80.5%)	59 711
Total	74 371 (29.4%)	178 736 (70.6%)	253 107

**Table 7:** Logistic regression for odds of severe pain during the last week as effect of diagnosis

Severe pain during last week in life, unadjusted logistic regression model					
		p value	OR	Lower 95% CI	Upper 95% CI
Diagnosis	Cancer	Ref.			
	Heart failure	< 0.001	0.51	0.49	0.53
	COPD	< 0.001	0.39	0.38	0.41
	Dementia	< 0.001	0.45	0.44	0.47
**Severe pain during last week in life, logistic regression model adjusted for sex and age**					
		p value	aOR	Lower 95% CI	Upper 95% CI
Diagnosis	Cancer	Ref.			
	Heart failure	< 0.001	0.60	0.57	0.62
	COPD	< 0.001	0.42	0.40	0.45
	Dementia	< 0.001	0.52	0.51	0.54
Sex	Women	Ref.			
	Men	0.033	1.02	1.00	1.04
Age	Continuous (per year)	< 0.001	0.99	0.99	0.99

## Discussion

In this nationwide, register based study we found that pain during the last week of life was registered in most patients dying of cancer (81%). It was also prevalent, although to a lower degree, in dementia (69%), heart failure (68%) and in COPD (57%). Severe pain was seen in 35% of all patients with cancer, and in 17–21% of patients with non-cancer conditions. Complete or partial pain relief was possible to obtain in most patients, as only 0,2% of the patients were not at all relieved.

### Cancer-related pain

In our study, we conclude that pain, as well as episodes of severe pain were more frequently seen in patients dying of cancer, than in heart failure, COPD or dementia, which is a reminder of why the modern hospice movement focused on the needs of cancer patients in the early days [21]. Our results, with more than 80% of patients having occurrence of pain during the last week of life, show higher prevalence than previous studies with 55% of patients having pain [12]. This difference might be affected by the fact that pain has been measured at different time points and pain seems to increase towards end of life [13]. Moreover, temporal patterns are of great importance, as the intensity of pain varies throughout the day and is affected by circumstances such as pain in rest compared to movement, which could affect the pain prevalence reported in different studies [22]. Within oncology, a phenomenon of breakthrough pain is referred to. As described in a recent study by Mercadante, breakthrough cancer-related pain is a complex phenomenon that may change its presentation during the course of patients’ disease [23], which might affect the prevalence of pain in different studies. Patients may experience breakthrough pain differently, so rather than defining breakthrough pain as a phenomenon with a typical pattern, it is likely that the plural term of “breakthrough pains” is more adequate [23]. Breakthrough of pain, despite ongoing pain treatment, is more common in cancer- than in non-cancer conditions. This is, however, not an excuse for inadequate pain management in non-cancer conditions. Clinically, it is more likely that staff asks a patient with advanced cancer about pain, compared to patients with non-cancer diagnoses, which leads to a risk of insufficient pain treatment in the latter groups.

### Pain in COPD and heart failure

The prevalence of pain in our study was 57% and 68% in COPD and heart failure, respectively. This is in agreement with other studies, although they show a considerable variation in figures from about 20 to 80% [24–29]. Reasons for different prevalence of pain in studies might be related to how pain is measured. Some studies only examine prevalence with a single yes/no question, while other studies rate pain intensities numerically or verbally and then report either all pain, including mild pain, or only moderate and severe pain [30]. Moreover, some studies use patient reported outcomes (PROs), whereas others rely on proxy measures, which may result in partly different figures [31, 32].

In COPD, some of the pain problems originate from the thorax [26], but also low back pain and chronic neck pain are common features [27]. In persons with heart failure, thoracic pain as well as abdominal pain originating from a swollen liver are commonplace [8]. Moreover, persons with COPD or hearth failure are often elderly, and in whom chronic muscle pain, as well as osteoarthritis are often seen [8]. In both groups, occurrence of pain was completely relieved in more than 70% of the cases in our study, and completely or partially relieved in more than 99% of the cases. However, the fact that 17% and 21% of the patients with COPD and hearth failure, respectively, had severe pain during the last week of life, shows that there is room for improvement, where an increased basic pain treatment would likely be most beneficial.

### Pain in dementia

Persons dying from dementia with concomitant pain problems need attention. Pain is in this group of patients known to be overlooked and, when diagnosed, also undertreated and more difficult to follow up [33]. When dementia is the main diagnosis, most persons eventually lose their ability to communicate, which requires the staff to use non-verbal instruments such as Abbey Pain Scale, to assess pain by the means of typical pain behaviors [34]. Compared with cancer, dementia is more seldom associated with pain problems, still pain was registered in as many as 69% of the patients in our study, which underlines that staff should be aware of the need of knowledgeable symptom control. Moreover, behavioral and psychological symptoms of dementia (BPSD) are often seen as the main challenge in dementia care, as BPSD affects patients, the families, and the staff. BPSD include a range of neuropsychiatric disturbances such as agitation, aggression, depression, and apathy. Recent studies have shown that BPSD might be associated with unrelieved pain, meaning that if pain is well controlled, also the problems with BPSD reduces significantly [35–37].

### Pain relief and opioid prescription

We found that he majority of patients with pain during the last week of life had complete relief of pain and most patients also had injectable opioids prescribed as needed. In Sweden, the goal is that all patients in end-of-life care have injectable opioids prescribed as needed, to enable good symptom control. Moreover, the Swedish National Board of Health and Welfare has defined prescription of injectable opioids in end-of-life care as a quality indicator, with a 98% target level as regards predictable deaths [38]. Thus, the high proportion of patients having pain relief and prescribed opioids as needed can be explained by these circumstances as pain relief is affected by the availability of opioids [39].

### Strengths and limitations

To the best of our knowledge, we here present the so far largest material on prevalence of pain during the last week of life, based on data from more than 300 000 persons. It is unique since pain was registered with the same questionnaire throughout, which makes the figures comparable between cancer and non-cancer diagnoses. While several prevalence studies are delimited to certain settings, e.g. to specialized palliative care, primary health care, nursing homes or acute hospital care [25, 26, 29], the prevalence of pain in this study is believed to have good potential for generalizability since our data cover all settings. Furthermore, the widespread use of the SPRC with its geographical representation from all parts of the country strengthens the external validity of our findings.

There are several limitations to our study. First, we do not know to what extent the underlying pain was preemptively treated, which may affect the proportion of people who experience significant pain. Further, adherence to agreed routines for data collection is a common problem for most registers, and the SRPC constitutes no exception [40]. Moreover, there are no instruments that are validated for symptom assessment in the imminently dying patient with lowered level of consciousness, which means that pain needs to be identified indirectly by the health care staff. Thus, when instruments were not used, the reporting was based on subjective judgments by the staff performing the registration. Moreover, pain assessment tools used in patients with dementia, such as the Abbey Pain Scale, might have inadequate psychometric properties in this population, such as validity, which can affect pain assessment negatively [41]. Consequently, symptom assessments to some degree depended on the level of knowledge, skills, and personal attitudes.

## Conclusions

The occurrence of pain, including severe pain is less common for both patients with heart failure, COPD and dementia, compared to patients with cancer. When present, pain is more often fully relieved for patients with dementia compared to cancer, but less often in heart failure and in COPD. As severe pain was seen in about a third of cancer patients, the study still underlines the need for better pain management in the imminently dying.

## Data Availability

The datasets generated, used, and analyzed during the current study available from the corresponding author on reasonable request.

## Abbreviations

aOR: adjusted Odds Ratio
BPSD: Behavioral and Psychological Symptoms of Dementia
CI: Confidence Interval
COPD: Chronic Obstructive Pulmonary Disease
ESAS: Edmonton Symptom Assessment Scale
IPOS: Integrated Patient care Outcome Scale
NRS: Numeric Rating scale
OR: Odds Ratio
PROs: Patient Reported Outcomes
SRPC: Swedish Register of Palliative Care
VAS: Visual Analogue Scale

## References

[1] Krikorian A, Maldonado C, Pastrana T (2020). Patient’s perspectives on the notion of a good death: a systematic review of the literature. J Pain Symptom Manag.

[2] Meier EA, Gallegos JV, Thomas LP, Depp CA, Irwin SA, Jeste DV (2016). Defining a good death (successful dying): Literature Review and a call for research and public dialogue. Am J Geriatr Psychiatry.

[3] Abbott CH, Prigerson HG, Maciejewski PK (2014). The influence of patients’ quality of life at the end of life on bereaved caregivers’ suicidal ideation. J Pain Symptom Manag.

[4] Henson LA, Maddocks M, Evans C, Davidson M, Hicks S, Higginson IJ (2020). Palliative Care and the management of common distressing symptoms in Advanced Cancer: Pain, breathlessness, nausea and vomiting, and fatigue. J Clin Oncology: Official J Am Soc Clin Oncol.

[5] Broese JM, de Heij AH, Janssen DJ, Skora JA, Kerstjens HA, Chavannes NH (2021). Effectiveness and implementation of palliative care interventions for patients with chronic obstructive pulmonary disease: a systematic review. Palliat Med.

[6] Sahlollbey N, Lee CKS, Shirin A, Joseph P (2020). The impact of palliative care on clinical and patient-centred outcomes in patients with advanced heart failure: a systematic review of randomized controlled trials. Eur J Heart Fail.

[7] Årestedt K, Brännström M, Evangelista LS, Strömberg A, Alvariza A (2021). Palliative key aspects are of importance for symptom relief during the last week of life in patients with heart failure. ESC Heart Fail.

[8] Alpert CM, Smith MA, Hummel SL, Hummel EK (2017). Symptom burden in heart failure: assessment, impact on outcomes, and management. Heart Fail Rev.

[9] Maddocks M, Lovell N, Booth S, Man WD, Higginson IJ (2017). Palliative care and management of troublesome symptoms for people with chronic obstructive pulmonary disease. Lancet.

[10] Kroenke K, Gao S, Mosesso KM, Hickman SE, Holtz LR, Torke AM (2022). Prevalence and predictors of symptoms in persons with Advanced Dementia living in the community. J Palliat Med.

[11] Bandeali S, des Ordons AR, Sinnarajah A (2020). Comparing the physical, psychological, social, and spiritual needs of patients with non-cancer and cancer diagnoses in a tertiary palliative care setting. Palliat Support Care.

[12] Snijders RAH, Brom L, Theunissen M, van den Beuken-van Everdingen MHJ. Update on Prevalence of Pain in patients with Cancer 2022: a systematic literature review and Meta-analysis. Cancers (Basel). 2023;15(3). 10.3390/cancers15030591 PMC9913127 36765547

[13] Seow H, Guthrie DM, Stevens T, Barbera LC, Burge F, McGrail K (2021). Trajectory of end-of-Life Pain and other physical symptoms among Cancer patients receiving Home Care. Curr Oncol.

[14] Elmstedt S, Mogensen H, Hallmans DE, Tavelin B, Lundström S, Lindskog M (2019). Cancer patients hospitalised in the last week of life risk insufficient care quality - a population-based study from the Swedish Register of Palliative Care. Acta Oncol.

[15] Conen K, Guthrie DM, Stevens T, Winemaker S, Seow H (2021). Symptom trajectories of non-cancer patients in the last six months of life: identifying needs in a population-based home care cohort. PLoS ONE.

[16] Klint Å, Bondesson E, Rasmussen BH, Fürst CJ, Schelin MEC (2019). Dying with unrelieved Pain-prescription of Opioids is not enough. J Pain Symptom Manag.

[17] Mercadante S (2018). Treating breakthrough pain in oncology. Expert Rev Anticancer Ther.

[18] Hagarty AM, Bush SH, Talarico R, Lapenskie J, Tanuseputro P (2020). Severe pain at the end of life: a population-level observational study. BMC Palliat Care.

[19] Osborne NR, Davis KD (2022). Sex and gender differences in pain. Int Rev Neurobiol.

[20] Lundström S, Axelsson B, Heedman PA, Fransson G, Fürst CJ (2012). Developing a national quality register in end-of-life care: the Swedish experience. Palliat Med.

[21] Clark D (2007). From margins to centre: a review of the history of palliative care in cancer. Lancet Oncol.

[22] Mercadante S, Bruera E. Different colors for breakthrough ESAS items. J Pain Symptom Manage. 2024. 10.1016/j.jpainsymman.2024.02.566 38447623

[23] Mercadante S (2023). Once again… breakthrough cancer pain: an updated overview. J Anesth Analg Crit Care.

[24] Moens K, Higginson IJ, Harding R (2014). Are there differences in the prevalence of palliative care-related problems in people living with advanced cancer and eight non-cancer conditions? A systematic review. J Pain Symptom Manag.

[25] Theander K, Hasselgren M, Luhr K, Eckerblad J, Unosson M, Karlsson I (2014). Symptoms and impact of symptoms on function and health in patients with chronic obstructive pulmonary disease and chronic heart failure in primary health care. Int J Chronic Obstr Pulm Dis.

[26] Janssen DJ, Wouters EF, Parra YL, Stakenborg K, Franssen FM (2016). Prevalence of thoracic pain in patients with chronic obstructive pulmonary disease and relationship with patient characteristics: a cross-sectional observational study. BMC Pulm Med.

[27] Fuentes-Alonso M, López-de-Andrés A, Palacios-Ceña D, Jimenez-Garcia R, Lopez-Herranz M, Hernandez-Barrera V (2020). COPD is Associated with higher prevalence of Back Pain: results of a Population-based case-control study, 2017. J Pain Res.

[28] Chaabouni M, Feki W, Moussa N, Bahloul N, Kammoun S (2022). Chronic Pain in patients with Chronic Obstructive Pulmonary Disease: A Cross Sectional Study. Tanaffos.

[29] Kernick L, Glare P, Hosie A, Chiu A, Kissane DW. Prevalence and management of chronic nonmalignant pain in palliative care populations: a systematic review. Palliat Support Care. 2023:1–7. 10.1017/S1478951523000378 37039456

[30] Busse JW, Bartlett SJ, Dougados M, Johnston BC, Guyatt GH, Kirwan JR (2015). Optimal strategies for reporting Pain in clinical trials and systematic reviews: recommendations from an OMERACT 12 workshop. J Rheumatol.

[31] Abahussin AA, West RM, Wong DC, Ziegler LE (2019). PROMs for Pain in Adult Cancer patients: a systematic review of Measurement Properties. Pain Pract.

[32] Murtagh FE, Ramsenthaler C, Firth A, Groeneveld EI, Lovell N, Simon ST (2019). A brief, patient- and proxy-reported outcome measure in advanced illness: validity, reliability and responsiveness of the Integrated Palliative care Outcome Scale (IPOS). Palliat Med.

[33] Achterberg W, Lautenbacher S, Husebo B, Erdal A, Herr K (2020). Pain in dementia. Pain Rep.

[34] Abbey J, Piller N, De Bellis A, Esterman A, Parker D, Giles L (2004). The Abbey pain scale: a 1-minute numerical indicator for people with end-stage dementia. Int J Palliat Nurs.

[35] Habiger TF, Flo E, Achterberg WP, Husebo BS (2016). The interactive relationship between Pain, psychosis, and agitation in people with dementia: results from a cluster-randomised clinical trial. Behav Neurol.

[36] Pergolizzi JV, Raffa RB, Paladini A, Varrasi G, LeQuang JA (2019). Treating pain in patients with dementia and the possible concomitant relief of symptoms of agitation. Pain Manag.

[37] Kaufmann L, Moeller K, Marksteiner J (2021). Pain and Associated Neuropsychiatric Symptoms in patients suffering from Dementia: challenges at different levels and proposal of a conceptual Framework. J Alzheimers Dis.

[38] The National Board of Health and Welfare. National guidelines – Target levels in Palliative care at the end of life, Target levels for indicators (Nationella riktlinjer – Målnivåer Palliativ vård i livets slutskede, Målnivåer för indikatorer). https://www.socialstyrelsen.se/globalassets/sharepoint-dokument/artikelkatalog/nationella-riktlinjer/2017-10-22.pdf; 2017.

[39] Hasegawa-Moriyama M, Morioka Y, Hiroi S, Naya N, Suzuki Y, Koretaka Y et al. High prevalence of severe pain is associated with low opioid availability in patients with advanced cancer: combined database study and nationwide questionnaire survey in Japan. Neuropsychopharmacol Rep. 2024. 10.1002/npr2.12448 PMC11544452 38735866

[40] Martinsson L, Heedman PA, Lundström S, Fransson G, Axelsson B (2011). Validation study of an end-of-life questionnaire from the Swedish Register of Palliative Care. Acta Oncol.

[41] Smith TO, Harvey K (2022). Psychometric properties of pain measurements for people living with dementia: a COSMIN systematic review. Eur Geriatr Med.

